# Incidence of CXCR4 tropism and CCR5-tropic resistance in treatment-experienced participants receiving maraviroc in the 48-week MOTIVATE 1 and 2 trials

**DOI:** 10.1177/2040206619895706

**Published:** 2019-12-19

**Authors:** Becky Jubb, Marilyn Lewis, Lynn McFadyen, Paul Simpson, Julie Mori, Phylinda Chan, Barry Weatherley, Elna van der Ryst, Mike Westby, Charles Craig

**Affiliations:** 1 Pfizer Inc, Clinical Group, Rare Disease, Groton, CT, USA; 2 The Research Network, Sandwich, UK; 3 Pfizer Inc, Pharmacometrics, Sandwich, UK

**Keywords:** AIDS, drug resistance, HIV, mutations

## Abstract

Maraviroc blocks HIV-1 entry into CD4+ cells by interrupting the interaction between viral gp120 and cell-surface CCR5. Resistance to CCR5 antagonist–mediated inhibition can develop by unmasking pre-existing CXCR4-using virus or through selection of CCR5-tropic resistant virus, characterized by plateaus in maximum percent inhibition <95%. Here, we examine viral escape in maraviroc-treated participants during virologic failure through Week 48 in the MOTIVATE 1 and 2 trials. Resistance was assessed relative to number of active drugs in participants’ optimized background therapy, pharmacokinetic adherence markers, Baseline demographic data, HIV-1 RNA and CD4+ counts. For participants with R5 virus confirmed (*post hoc*) at Screening, Baseline genotypic weighted optimized background therapy susceptibility scores (gwOBTSS) were assigned where possible. Through Week 48, 219/392 (56%) participants with an assigned gwOBTSS achieved a virologic response. Of those remaining, 48/392 (12%) had CXCR4-using virus; 58/392 (15%) had R5 virus (maraviroc sensitive: *n* = 35/392, 9%; maraviroc resistant: *n* = 18/392, 5%; undeterminable: *n* = 5/392, 1%) and 67/392 (17%) had no failure tropism result. When optimized background therapy provided limited support to maraviroc (gwOBTSS <2), 143/286 (50%) responded to therapy, while 76/106 (72%) participants with gwOBTSS ≥2 responded (*p* < 0.001). Resistance rates were highest for participants with gwOBTSS <2, accounting for 45/48 (94%) of total CXCR4-using emergence and 18/18 (100%) of total CCR5-tropic resistance. R5 viruses from participants with gwOBTSS ≥2 (*n* = 10) were exclusively maraviroc sensitive; five of these participants had pharmacokinetic and/or pill-count markers of non-adherence. When co-administered with a fully active background regimen, maraviroc did not readily generate resistance in the clinical setting.

**Trial registry name:**

ClinicalTrials.gov (https://clinicaltrials.gov/), NCT00098722 and NCT00098306

## Introduction

Maraviroc is a first-in-class CCR5 antagonist currently approved in the United States for twice daily (BID) use in CCR5-tropic (R5) HIV-1-infected adults, adolescents and children at least 2 years old who weigh at least 10 kg, (https://www.accessdata.fda.gov/drugsatfda_docs/label/2016/208984_022128s017lbl.pdf) and in Europe for antiretroviral treatment–experienced persons of the same age and weight group (http://www.ema.europa.eu/docs/en_GB/document_library/EPAR_-_Product_Information/human/000811/WC500022190.pdf). In the Phase III MOTIVATE 1 and 2 studies, maraviroc (once daily [QD] or BID) plus optimized background therapy (OBT) demonstrated significantly greater virologic and immunologic efficacy and a similar safety profile compared with placebo plus OBT at Week 48 in treatment-experienced participants with R5 virus (as identified using the original Trofile® assay [Monogram Biosciences, South San Francisco, CA, USA]).^1,2^ All participants receiving maraviroc QD were offered maraviroc BID when the last patient had reached Week 48 and the study was unblinded; overall responses were then durable with maraviroc BID through to Week 96.^3^

In contrast to other antiretroviral drugs, maraviroc resistance does not manifest itself in phenotypic drug susceptibility assays as a shift in half-maximal inhibitory concentration (IC_50_) following serial *in vitro* passage of virus with drug.^4^ This is thought to be a consequence of maraviroc’s novel mode of action, which involves binding to a host cell receptor rather than to a viral component.^5,6^ Maraviroc binding to CCR5 provokes an allosteric conformational change, which inhibits viral engagement with the coreceptor and prevents entry. Thus, rather than mutating to prevent the drug from binding to its target, maraviroc resistance arises when the virus mutates to make use of drug-bound CCR5. This is characterized phenotypically in drug susceptibility assays by reduced maximum percent inhibition (MPI) in which 100% inhibition of viral replication is not achieved even at saturating drug concentrations.^4^ Such a mechanism has also been described for other CCR5 antagonists^7,8^ and was confirmed for maraviroc in a pre-planned interim analysis of a subset of MOTIVATE participants.^9^

Detailed analysis of viruses obtained from participants enrolled in the MOTIVATE studies demonstrated that virologic failure in participants receiving maraviroc-containing regimens can occur either as a result of ‘un-masking’ of pre-existing CXCR4-using virus (CXCR4- or dual-mixed tropic)^10^ or with R5 virus, which may or may not harbor phenotypic resistance to maraviroc.^4,11–13^

As with other studies in treatment-experienced participants,^14,15^ pre-planned subgroup analyses of the MOTIVATE studies demonstrated that more participants who received a higher number of potentially active drugs in their OBT according to Baseline susceptibility scores achieved virologic suppression.^1^ Subsequent *post hoc* analyses of the MOTIVATE virologic population have shown that an alternative methodology, using a genotypic weighted OBT susceptibility score (gwOBTSS), provides a more relevant measure of the contribution of the OBT to the overall regimen antiviral activity than simply counting active drugs. It also shows a significant correlation with treatment outcome.^16^

In the present study, we describe for the first time the maraviroc tropism and R5 virus resistance findings for the MOTIVATE studies at Week 48, the time of the protocol-defined primary endpoint analysis, using the enhanced sensitivity Trofile assay (ESTA)-derived population. In addition, the incidence of maraviroc resistance in the MOTIVATE trials is reported and discussed in the context of activity of the OBT.

## Methods

### Study population

MOTIVATE 1 and MOTIVATE 2 were parallel, randomized, double-blind, placebo-controlled multinational Phase III studies.^1,2^ The two studies differed only by geographic location, and all participants were infected with R5 HIV-1 at Screening, as determined using a co-receptor tropism assay (original Trofile assay), and had taken one or more agents from three antiretroviral classes for six months or had documented genotypic or phenotypic resistance to drugs from at least three classes of antiretroviral drug. Maraviroc (150 mg QD or BID) or placebo was administered along with an optimized background of antiretroviral drugs (i.e. OBT) based on treatment history and drug resistance testing.

The primary endpoint of the MOTIVATE 1 and 2 trials was the mean change in plasma HIV-1 RNA concentration (log_10_ copies/mL) from Baseline.

In the current analysis, virologic failure was defined using time to loss of virologic response criteria including failure to achieve plasma HIV-1 RNA <50 copies/mL (TLOVR50) by Week 48, discontinuation prior to Week 48 for lack of efficacy or two consecutive plasma HIV-1 RNA measurements ≥50 copies/mL occurring after a confirmed <50 copies/mL result. Participants who continued therapy through Week 48 without meeting any of the above failure definitions were considered responders. Failure tropism and maraviroc susceptibility were assessed at discontinuation or Week 48, the primary endpoint visit.

The study protocols were approved by the institutional review board or independent ethics committee at each study center (see Supplemental Table 1 for a complete list). Written informed consent was obtained from all participants. The studies were performed in accordance with International Conference on Harmonisation Good Clinical Practice guidelines and applicable local regulatory requirements and laws. The studies were registered with ClinicalTrials.gov (identifiers: NCT00098722 and NCT00098306).

### HIV tropism and maraviroc susceptibility determination

Tropism was determined from virus in plasma samples using the Trofile assay. Possible results included CCR5-tropic (R5), CXCR4-tropic (X4) and dual- or mixed-tropic (DM) virus. Dual-tropic and mixed-tropic viruses cannot be distinguished in this assay. X4 and DM results may be classified together as CXCR4-using virus. Enrolment into the MOTIVATE 1 and 2 trials required participants to be infected with R5 HIV-1, as determined using the original Trofile assay. However, after conclusion of the trials, a more sensitive method for detection of CXCR4-using virus became available (ESTA)^17^ and a retrospective reanalysis of Screening tropism was performed to identify participants with detectable, pre-existing CXCR4-using virus. These participants would not have been enrolled if ESTA had been available at the time of the trials.

Maraviroc susceptibility was determined by Monogram Biosciences using the PhenoSense® HIV-1 Entry assay and was described in terms of both IC_50_ fold change (FC) and MPI. Resistance was defined as virus with MPI <95%, based on previous findings with *in vitro* generated maraviroc-resistant viruses^4,18^ and studies of participants failing early in the MOTIVATE trials.^9^

### Susceptibility score determination

Sequencing of the reverse transcriptase and protease at Screening was performed by Monogram Biosciences. Enfuvirtide was genotypically assessed at the BC Centre for Excellence in HIV/AIDS, Vancouver, British Columbia, Canada. Genotypic susceptibility scores and overall susceptibility scores were provided by Monogram Biosciences, who used their proprietary algorithms.

The gwOBTSS was determined as described previously.^16^ In brief, the results were interpreted using the French National Agency for AIDS Research (ANRS) algorithm (ANRS-AC 11: Resistance group, July 2008, version n°17) and, together with treatment history, were used to determine gwOBTSS. Drugs in continuous use pre-screening through Week 48 or earlier discontinuation were not counted, as drugs already present in a prolonged, stable, failing regimen pretreatment do not contribute to post-Baseline responses. In addition, based on data showing that active nucleosides contribute only approximately 50% of the virologic activity of drugs from other classes in treatment-experienced participants,^19^ active nucleos(t)ide reverse-transcriptase inhibitors (NRTIs) were assigned a score of 0.5. Protease inhibitors (PIs) with an ‘intermediate’ resistance genotype scored 0.5 and other active agents scored 1.0.

The ANRS algorithm has since been updated (ANRS-AC 11: Resistance group, September 2017, version n°27). The effects of this change on the gwOBTSS analysis was determined for participants included in the analysis of the relationship between the gwOBTSS and maraviroc susceptibility, with no changes in gwOBTSS among the 53 participants.

### Pharmacokinetic measurements

Sparsely sampled random pharmacokinetic (PK) measurements (1–2 per visit) were performed during visits for the first 24 weeks of therapy. Samples with maraviroc concentrations below the limit of quantification (0.5 ng/mL; BLQ) were considered to display a marker of poor adherence.^20^

### Statistical analysis

Differences between maraviroc log_10_ IC_50_ FC of R5 viruses at Baseline and failure time points were tested using a paired *t*-test. To compare maraviroc MPIs, the Wilcoxon matched pairs test was employed. Differences in treatment outcomes and failure tropism between gwOBTSS groups and treatment arms were analyzed using a Chi-squared test. Odds ratios and Wald 95% confidence intervals (CIs) were calculated by dichotomizing the variables. While medians were used for age (<43 vs. ≥43 years) and time since diagnosis (<13 vs. ≥13 years), for CD4 and HIV-1 RNA, 100 cells/µL and 100,000 copies/mL, respectively, were used as the cut-offs. A continuity correction of 0.5 was applied for calculating the odds ratios.

## Results

### Virologic outcomes and gwOBTSS participant populations

**Figure 1.**
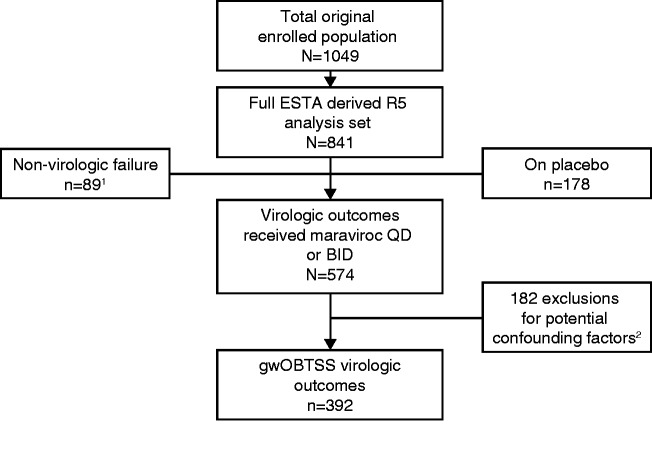
Participant accountability. BID, twice daily; ESTA, enhanced sensitivity Trofile assay; gwOBTSS, genotypic weighted optimized background therapy susceptibility score; OBT, optimized background therapy; QD, once daily. 1. Includes adverse events *n* = 31, default *n* = 37, death *n* = 6 and other *n* = 15. 2. Exclusions were based on the same criteria as described previously.^16^ Briefly, these include Baseline visit >7 days before start of randomized therapy (*n* = 3), OBT changes while on-treatment or drug use changes between the Screening and OBT windows (*n* = 129), OBT drugs that had an interruption of treatment that could have resulted in an incorrect gwOBTSS score (*n* = 22) and unavailable resistance test result for an OBT drug (*n* = 34). More than one event could have occurred with any given participant. The OBT window was a period of seven days either side of the ‘Day 1’ Baseline study visit for the OBT to be initiated.

Of 841 participants enrolled in the MOTIVATE 1 and 2 trials with R5 virus at Screening as assessed using ESTA, 663 received at least one dose of maraviroc (Figure 1).

To focus on the mechanisms associated with virologic failure, 89 participants who discontinued for non-virologic reasons were excluded from the analysis to give a total of 574 maraviroc recipients with a known virologic outcome at Week 48 (the Virologic Outcomes Population). To study participants who had a consistent OBT through Week 48, only participants to whom a gwOBTSS could be assigned were included in the current analysis (392/574, 68%, the gwOBTSS Virologic Outcomes Population). Failure to assign a gwOBTSS score was due to potential confounding factors, the most common of which was a change in OBT during blinded therapy (*n* = 129) (Figure 1). The total Virologic Outcomes placebo group consisted of 178 participants with R5 virus at Screening, established using the ESTA.

### Participant characteristics

Baseline characteristics of the 574 maraviroc-treated participants with virologic outcomes and the 392 participants with a valid gwOBTSS were similar to those of the entire cohort of 1049 enrolled participants^2^ and were balanced between treatment groups (Table 1).

**Table 1.** Baseline demographic characteristics of participants studied in this sub-analysis compared with the full participant population for the MOTIVATE trials and the gwOBTSS Virologic Outcomes Population.

	Virologic Outcomes Population *n* = 574	gwOBTSS Virologic Outcomes		Full participant population *n* = 1049
		Population *n* = 392	Exclusions *n* = 182	
Gender, male %	88	88	90	89
Median age (yr)	45 (19–75)	45 (21–73)	46 (19–75)	46 (17–75)
Median plasma HIV-1 RNA log_10_ copies/mL	4.86 (3.31–6.92)	4.83 (3.55–6.92)	4.93 (3.31–5.98)	4.86 (3.31–7.09)
Median Baseline CD4 count cell/mm^3^	178.5 (1.5–965.5)	182.8 (2.0–965.5)	175 (1.5–914)	169 (0.5–965.5)

gwOBTSS: Genotypic weighted optimized background therapy susceptibility score.

### HIV-1 tropism and R5 susceptibility at virologic failure

A treatment response (HIV-1 RNA <50 copies/mL at Week 48) was observed at Week 48 in 311 of the 574 (54%) participants in the Virologic Outcomes Population, and a further 81 (14%) participants had insufficient plasma HIV-1 RNA (<500 copies/mL) for tropism testing. CXCR4-using virus was detected at failure or Week 48 in 75 (13%) participants; 87 (15%) participants failed with R5 virus and 20 (3%) participants had a non-reportable tropism result. Of the 87 participants failing with R5 virus, virus from 53 (61%) showed maraviroc sensitivity, 26 (30%) had selected resistance to maraviroc and 8 (9%) did not have both Baseline and failure maraviroc susceptibility data available.

When the two maraviroc treatment arms (QD and BID) were compared, there was no significant difference in the distribution of outcomes or viral tropism at failure between the BID and QD arms (*p* = 0.13, Chi-square test), and the proportion of evaluable R5 viruses with maraviroc resistance was comparable between treatment arms at failure (*p* = 0.82, Chi-square test).

**Figure 2.**
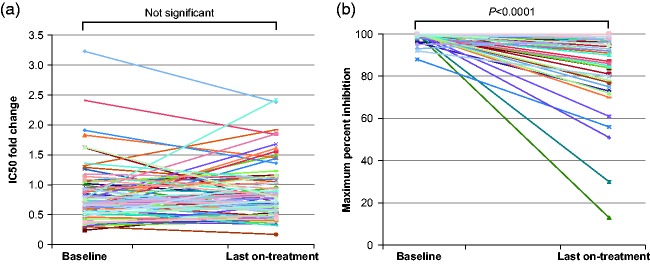
Sensitivity of R5 viruses to maraviroc at Baseline and at the last on treatment determination at TLOVR50 failure. Sensitivity assessed by (a) maraviroc IC_50_ compared with reference strain (JRCSF) (excludes two participants whose virus was not inhibited to 50% at the highest concentration of maraviroc used in the assay), and (b) maraviroc MPI. Differences between log_10_ IC_50_ FCs tested using paired *t*-test. Differences between MPIs tested using the Wilcoxon matched pairs test. FC: fold change; IC_50_: half-maximal inhibitory concentration; MPI: maximum percent inhibition; TLOVR50: time to loss of virologic response criteria including failure to achieve plasma HIV-1 RNA <50 copies/mL.

In the group of 79 participants experiencing failure with R5 virus and with successful paired Baseline and failure maraviroc susceptibility determinations, there was no difference between IC_50_ FC at Baseline and failure (geometric mean IC_50_ FC Baseline: 0.75, 95% CI: 0.67–0.84; failure: 0.78, 95% CI: 0.69–0.87, *p* = 0.5, paired *t*-test; Figure 2(a)) . However, a significant difference was observed between maraviroc MPI at Baseline and failure (MPI mean ± standard deviation: Baseline: 99.3 ± 1.9, range 88–100%; failure: 91.3 ±15.8, range 13–100%, *p* < 0.0001, Wilcoxon matched pairs test; Figure 2(b)).

In a sensitivity analysis, a snapshot, virology-first approach was used to identify per-protocol participants who did not have <50 copies/mL at Week 48 and who did not discontinue treatment because of adverse events or death. These were assessed for tropism and maraviroc susceptibility at failure. Of 243 virologic failures, 151 had valid tropism determinations at failure; of these, 67 were CXCR4-using and 84 had R5 virus. Seventy of those with R5 virus had maraviroc susceptibility data, and of these, 24 showed resistance. These values were comparable to those for the analysis based on TLOVR50 failure.

In the total Virologic Outcomes placebo group, 122 of 178 participants (69%) showed virologic failure; 113 (63%) had >500 copies/mL plasma HIV-1 RNA and were eligible for tropism assessment: 7 (6.2%) had CXCR4-using tropism, 8 (7.1%) failed tropism testing, and 98 (87%) had R5 virus at failure. Twenty-one of these 98 participants had maraviroc susceptibility data, one of whom showed resistance with an MPI of 41%.

### Baseline resistance to maraviroc

Phenotypic resistance to maraviroc was identified at Baseline in three participants in the Virologic Outcomes Population who later experienced failure with R5 virus. Two of these participants had Baseline MPIs of 92% and 88%, which further reduced during therapy to 80% and 56%, respectively. Virus from the remaining participant had a Baseline MPI of 93% but maraviroc-sensitive virus (MPI >95%) was found at failure.

### Treatment outcomes for participants within the gwOBTSS virologic outcomes population

**Figure 3.**
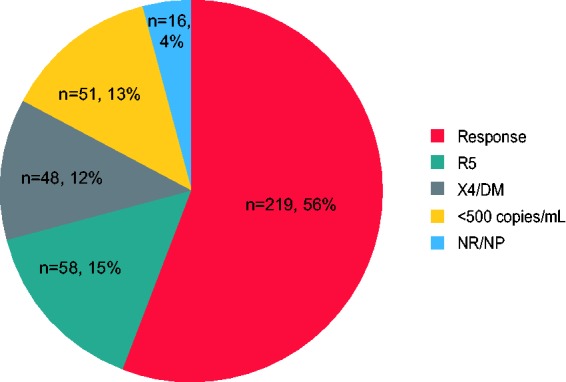
TLOVR50 outcome and viral tropism at failure. This analysis was performed with the ESTA-derived gwOBTSS Virologic Outcomes Population (*n* = 392). R5: failure with R5-tropic virus; X4/DM: failure with CXCR4-using virus; <500 copies/mL: failure with HIV-1 plasma RNA below cut-off for tropism determination; NR/NP: tropism result not available. ESTA: Enhanced Sensitivity Trofile Assay; gwOBTSS: genotypic weighted OBT susceptibility score; TLOVR50: time to loss of virologic response criteria including failure to achieve plasma HIV-1 RNA <50 copies/mL.

A significant association between virologic response and the Baseline gwOBTSS and CD4 counts was previously demonstrated in a *post hoc* analysis of MOTIVATE 1 and 2.^16^ To examine the relationship between failure with maraviroc-resistant R5 virus and Baseline factors, a retrospective analysis was performed using the ESTA-derived gwOBTSS Virologic Outcomes Population. Proportions of participants with each outcome were consistent with the Virologic Outcomes Population described above. Just over half of participants (219/392, 56%) responded to treatment through Week 48; 58 of 392 (15%) participants failed with R5 virus and 48 of 392 (13%) participants failed with X4 or DM virus (Figure 3). In the original gwOBTSS Virologic Outcomes Population sub-group (i.e. that included participants with CXCR4-using virus detected at Screening using ESTA) (*n* = 493), a slightly lower proportion of participants responded to treatment (260/493, 53%), with a corresponding increase in the proportion of participants experiencing failure with X4 or DM virus (*n* = 91/493, 18%) (Supplemental Figure 1).

### Relationship between virologic outcome and activity of the background regimen

**Figure 4.**
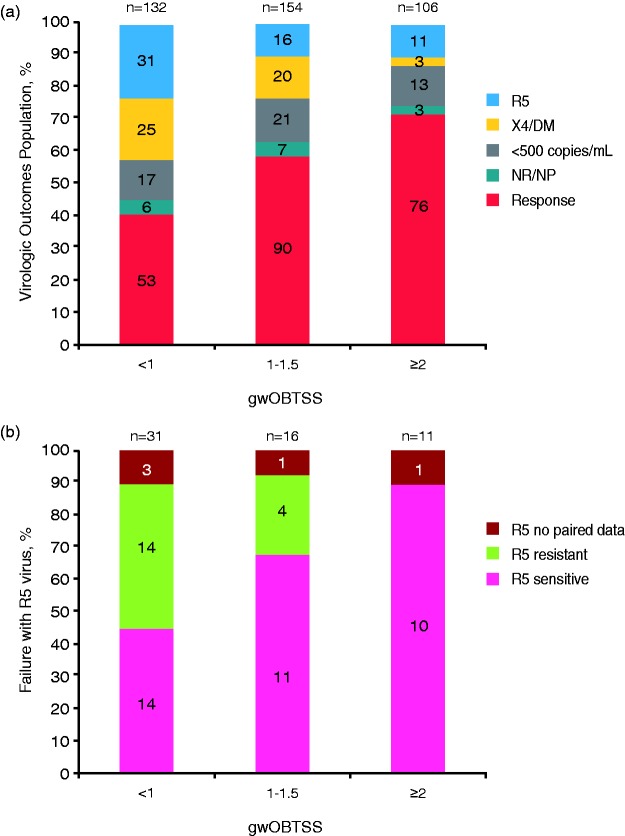
Outcomes, (a) failure tropism and (b) R5 maraviroc susceptibility in the ESTA-derived gwOBTSS Virologic Outcomes Population by gwOBTSS. R5: failure with R5-tropic virus; X4/DM: failure with CXCR4-using virus; <500 copies/mL: failure with HIV-1 plasma RNA below cut-off for tropism determination; NR/NP: tropism result not available. ESTA: Enhanced Sensitivity Trofile Assay; gwOBTSS: genotypic weighted OBT susceptibility score.

The relationship between gwOBTSS and TLOVR50 outcome at Week 48 in the 392 participants in the ESTA-derived gwOBTSS Virologic Outcomes Population was assessed. Significantly more participants with gwOBTSS ≥2 responded to the maraviroc-based regimens (76/106, 72%) than in those with less background antiretroviral activity (gwOBTSS <2: 143/286, 50%; *p* < 0.001, Chi-square test). Further breakdown of gwOBTSS groups confirmed the increase in response with gwOBTSS (gwOBTSS: <1: 53/132, 40%; 1–1.5: 90/154, 58%; ≥2: 76/106, 72%, *p* < 0.0001, Chi-square test) (Figure 4(a)). There was no significant difference in the distribution of outcomes between the QD and BID treatment arms.

CXCR4-using failure accounted for 25/132 (19%), 20/154 (13%) and 3/106 (3%) of the population in the <1, 1 to 1.5 and ≥2 gwOBTSS groups, respectively, and R5 failure accounted for 31/132 (23%), 16/154 (10%) and 11/106 (10%) of the population across these gwOBTSS groups, respectively. The proportion of R5 viruses with phenotypic resistance to maraviroc at failure changed significantly with gwOBTSS (*p* = 0.01, Chi-square test). Indeed, 45/48 failures with CXCR4-using virus and 18/18 failures with R5-viral resistance on failure had regimens with gwOBTSS <2 (Figure 4). There was no significant difference in the proportion of failure with maraviroc-resistant virus between the QD and BID treatment arms.

A similar pattern was observed in the original Trofile-derived gwOBTSS Virologic Outcomes Population with the proportion of participants responding to therapy increasing with gwOBTSS and failure with R5-tropic resistance being most prevalent in participants with a gwOBTSS of 0 to 0.5 and not observed at gwOBTSS ≥2 (Supplemental Figure 2).

### Participants failing with maraviroc-susceptible virus

Additional analyses were performed to assess why failure occurred in participants with virus that was fully sensitive to maraviroc (*n* = 35), including 10 with gwOBTSS ≥2. One possible explanation was poor adherence to therapy. The absence of detectable plasma maraviroc, pill count and the variability of the modeled PK parameters over the period of sparse sampling (through Week 24) was assessed for evidence of poor adherence to maraviroc. For this analysis, 6 of the 35 participants were excluded due to the absence of PK data at failure (five of seven with rebound failed after Week 24; one other did not have a plasma maraviroc concentration available). Of the remaining 29 participants with maraviroc-sensitive R5 virus at failure, 15 were found with evidence of poor adherence including five of eight (62.5%) who received a regimen with optimal antiretroviral activity (gwOBTSS ≥2), five of nine (55.6%) with gwOBTSS of 1 to 1.5, and 5 of 12 (41.7%) received a weak or inactive regimen (gwOBTSS <1).

### Comparison between participants failing with maraviroc-susceptible or -resistant R5 virus

**Figure 5.**
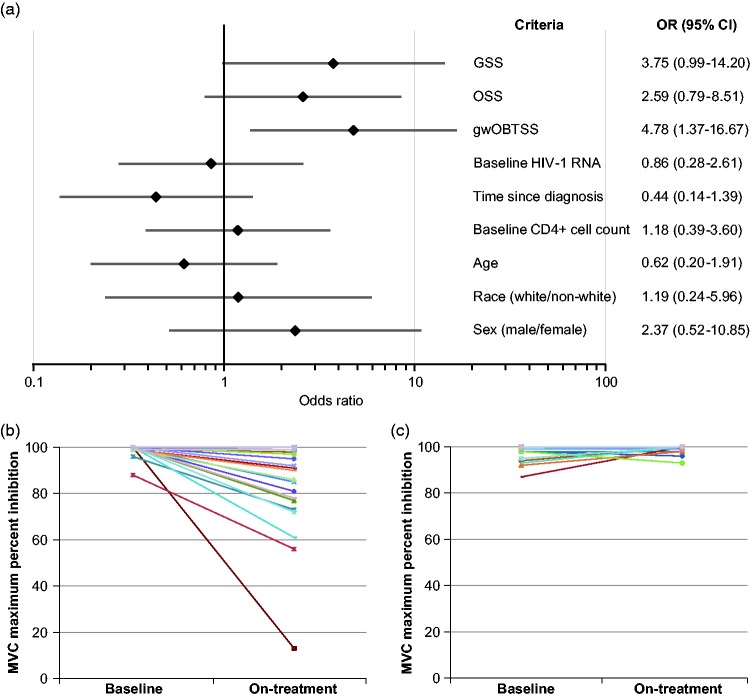
Odds ratio determinations for demographic, pharmacokinetic, and virologic characteristics of participants with virologic failure with R5 virus. (a) Odds ratios and 95% CIs for prediction of resistance among R5 failures. (b) Change in MPI between Screening and failure in participants treated with gwOBTSS ≤0.5. (c) Change in MPI between Screening and failure among participants with gwOBTSS >0.5. Odds ratios were calculated based on high and low values based on > or ≤ median values for continuous variables or on stratification criteria (CD4 cell count: 100 cells/mm^3^; HIV-1 RNA: 100,000 copies/mL; age: 43 years old; time since diagnosis: 13 years; GSS and OSS: 2; gwOBTSS: 0.5). A continuity correction of 0.5 was applied. CI: confidence interval; GSS: genotypic susceptibility score; gwOBTSS: genotypic weighted OBT susceptibility score; MPI: maximum percent inhibition; OSS: overall susceptibility score.

To further explore the reasons for failure with R5 virus with resistance, an analysis of potential parameters associated with R5 failure having maraviroc resistance was performed (Figure 5).

None of the demographic or PK parameters shown in Figure 5 produced any odds ratios indicating a significant relationship between these and the outcome of virologic susceptibility to maraviroc. The only significant finding was that of the gwOBTSS (OR, 4.78 [95% CI, 1.37–16.67] for resistance with gwOBTSS <1.0). In addition, resistance was observed only in instances where there was always detectable maraviroc in plasma when sparse sampling was performed (17/17). Poor adherence, as assessed by the absence of detectable maraviroc in plasma on sparse sampling, was found in 7/34 participants failing with maraviroc-susceptible R5 virus.

## Discussion

Previously we have examined in detail an interim data set based on a Week 24 data cut for the first 267 participants enrolled in the MOTIVATE trials.^9^ In the current analysis, only viruses from participants with R5 viral infections at Screening as determined using ESTA were included for the whole clinical data set through Week 48, the time of the primary endpoint analysis. The earlier analysis allowed for a more detailed clonal analysis in a smaller number of participants to establish principles of tropism pre-existence (*n* = 20) and to further examine mutations that might be associated with resistance to maraviroc in R5 virus (*n* = 4).^9^ The current analysis differs in that it describes the incidence of maraviroc resistance in the larger Week 48 data set (48 failures with CXCR4 virus and 58 with R5 virus, 18 of which showed maraviroc resistance in the maraviroc-treated groups) and examines potential clinical correlates of maraviroc resistance.

Another study examined the correlates of virologic failure in relation to the background regimen activity and other clinical parameters using the data from the population identified with the original screening Trofile assay.^16^ The current *post hoc* analysis expands on that analysis to describe the incidence of R5-tropic maraviroc resistance in the participants included in the ESTA-derived MOTIVATE populations who experienced virologic failure and further explores potential correlates of such resistance. Overall, 56% of participants with a virologic outcome for whom a valid gwOBTSS could be assigned achieved a TLOVR50 response through Week 48. Twelve percent of participants had CXCR4-using virus detected at the time of failure; 15% of the population failed with R5 virus and 31% of these had maraviroc-resistant R5 virus detected, the majority of whom had gwOBTSS 0 to 0.5. Findings were similar when failure was identified using either TLOVR50 or snapshot criteria.

It has been suggested that the proportion of participants failing with CXCR4-using virus in the MOTIVATE studies was higher than that observed in trials of another CCR5 antagonist, vicriviroc, in treatment-experienced participants.^21^ The current MOTIVATE re-analysis demonstrates that retrospective exclusion of participants with CXCR4-using virus detected using ESTA at Screening does indeed decrease the proportion of CXCR4-using viruses observed at treatment failure with maraviroc. The majority of the remaining participants who went on to have detectable CXCR4-using virus at failure had weakened support from their OBTs. Such participants represented approximately twice the proportion of the MOTIVATE study population compared with that in the vicriviroc Phase III VICTOR study, in which an overall greater proportion of participants had fully active antiviral support from the OBT (OBT fully active drugs, unweighted: ≤2: MOTIVATE: 72%–75%; VICTOR: 36%).^1,22^

Consistent with maraviroc’s mode of action, and as previously reported for *in vitro* generated maraviroc-resistant viruses and in other clinical studies with other CCR5 antagonists,^8,21,23^ resistance to CCR5 antagonists was characterized by a significant reduction in MPI rather than an increase in IC_50_ FC. Binding of maraviroc to the host protein, CCR5, rather than to a viral target, results in the virus becoming resistant via mutations that promote entry through gp120 recognition of the drug-bound receptor. As there is no dependency on the virus for drug binding, the concentration required to achieve full occupancy of cell-surface CCR5, when susceptible virus would show 100% inhibition, remains unaltered. However, even with full occupancy, resistant virus can achieve entry, albeit with reduced efficiency, and so the degree of inhibition is less than 100%.

Antiviral activity of the background drugs in the participant’s regimen was reported previously as an important determinant of treatment outcome in this highly treatment-experienced population.^16^ While maraviroc provided benefit when added to a regimen with any gwOBTSS, the correlation between gwOBTSS and response rate suggests that optimal benefit is obtained from its addition to regimens while options for optimization with fully active drugs remain.

Consistent with this, most participants who failed therapy with maraviroc-resistant R5 virus had gwOBTSS <1, and so the main selective pressure exerted by their treatment regimen was from maraviroc with little or no support from other drugs. These findings are similar to those from trials with vicriviroc,^21,24^ in which four of five treatment-experienced participants with vicriviroc-resistant R5 virus had an overall susceptibility score of 0, and with raltegravir,^15^ where the majority of integrase mutations were identified in virus from participants failing treatment with a genotypic or phenotypic susceptibility score to their OBT of 0. Under conditions of functional monotherapy, any maraviroc-resistant virus emerging would be expected to become the dominant species as it replicates in the presence of maraviroc-selective pressure, unchecked by other components of the treatment regimen. However, the finding that a third of participants treated with functional maraviroc monotherapy successfully achieved and maintained viral suppression demonstrates sufficient antiretroviral activity in these individuals to reduce viral replication to levels where resistance to maraviroc is not readily generated, even in the absence of an active OBT.

In contrast, maraviroc-resistant R5 viruses were not detected in any participants with gwOBTSS ≥2. In this group, where effective treatment was achieved with regimens including at least three active antiretroviral drugs, several drugs with varying modes of action maintained viral suppression sufficiently to prevent resistance arising and 72% of participants achieved sustained viral suppression. Plasma maraviroc concentrations were available for eight participants in this group who also failed with maraviroc-sensitive R5 virus. Periods of incomplete adherence to maraviroc therapy could be identified in all but one participant. During such periods, a lack of drug pressure provides an opportunity for the replication of drug-sensitive virus.

The study is limited by the diversity of the treatments employed, which was required because of the advanced disease stage and degree of treatment experience in the study population. Because the more recently developed integrase strand transfer inhibitors as well as darunavir were not approved for use at the time of the study, there were limitations on the treatment options available to participants. This will have contributed to the low weighted scoring of antiretroviral activity in the regimens, especially whenever continued use of a drug in enrollees occurred after prolonged use on a failing regimen. Difficulties in maintaining advanced-stage participants on one background regimen also contributed to the number of confounding data sets. Despite this, the results reinforce the importance of an adequately active background regimen and patient adherence in the treatment of HIV.

## Conclusions

In summary, failure of maraviroc-based therapy with maraviroc-resistant R5 virus was uncommon with the incidence related inversely to the number of active drugs in the participants’ background regimens. Maraviroc as functional monotherapy or with weak antiviral support from the OBT accounted for approximately 50% of CXCR4-using failure and 80% of R5 phenotypic resistance observed in the MOTIVATE gwOBTSS Virologic Outcomes Population. In the presence of a fully active background therapy (gwOBTSS ≥2), reduced proportions of CXCR4-using viruses were observed and R5 failure was exclusively maraviroc sensitive and related to markers of non-adherence to therapy.

## Data Availability

Anonymized individual participant data and study documents can be requested for further research from www.clinicalstudydatarequest.com.
